# Supplementary material for: Comparing the catch composition, profitability and discard survival from different trammel net designs targeting common spiny lobster (Palinurus elephas) in a Mediterranean fishery
Source: PeerJ. 2018 May 17;6:e4707. doi: 10.7717/peerj.4707 (PMC5960583; doi:10.7717/peerj.4707)
Supplement: Supplemental Information 1 [file peerj-06-4707-s001.docx]

**SUPPLEMENTAL INFORMATION**

**TableS1**. Description of the codes used to score the vitality of captured fishes (*Benoit, Hurlbut & Chasse, 2010*)

| **Vitality** | **Code** | **Description** |
| --- | --- | --- |
| Excellent | 1 | Vigorous body movement; no or minor external injuries only |
| Good/fair | 2 | Weak body movement; responds to touching/prodding; minora external injuries |
| Poor | 3 | No body movement but fish can move operculum; minor or major external injuries |
| Moribund | 4 | No body or opercular movements (no response to touching or prodding) |

**FigureS1. Autocorrelation plot.**

**
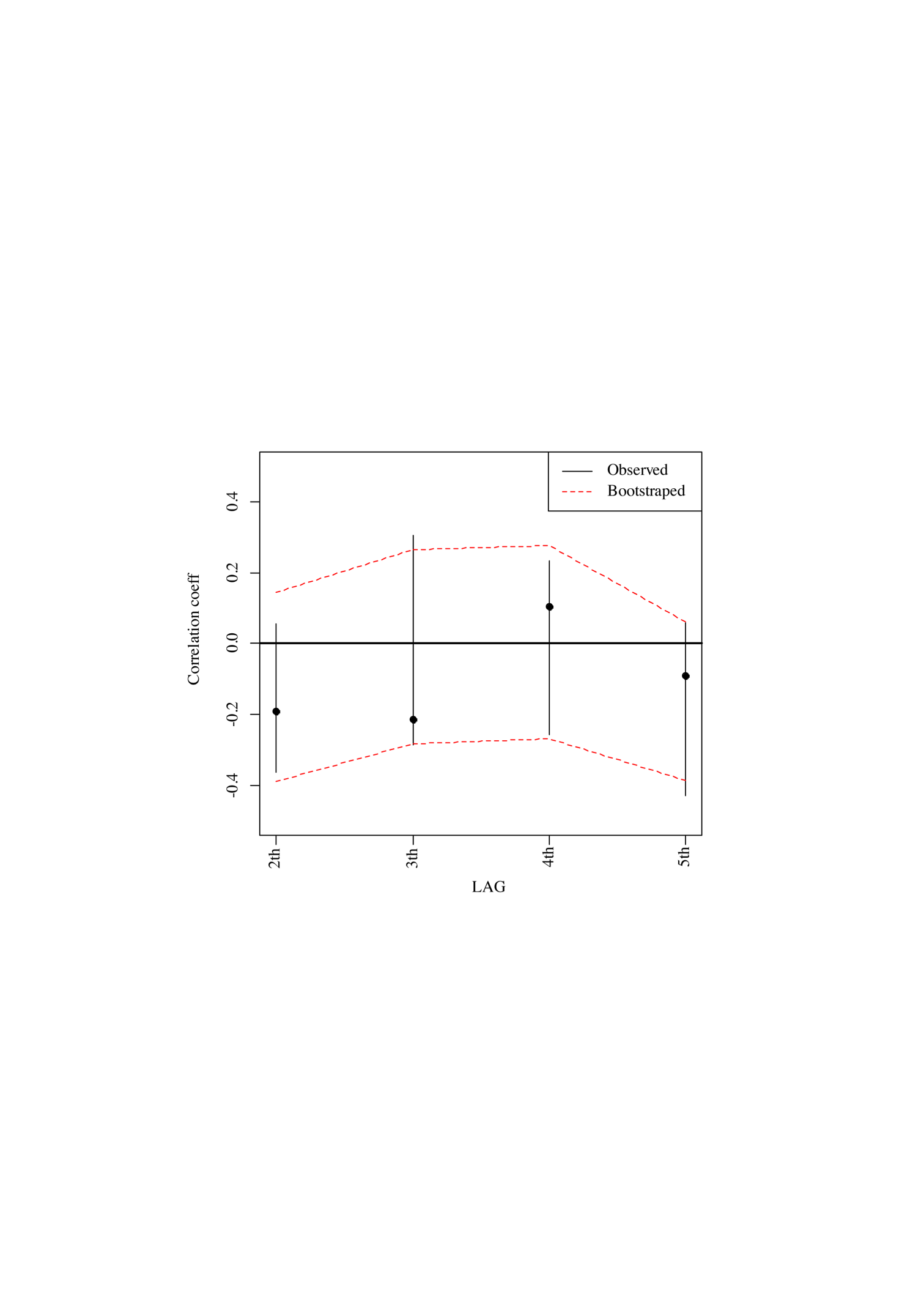
**

For the second lag, the residual of the netting wall at the i position are compared with the same residuals but at the i+1 position (i.e., with the closest neighbour, in the net). For the 3th, 4th and 5th lag, the netting wall at the i position are compared with the same residuals but at the i+2, i+3 and i+4 position. The actually observed correlation (and its 95% confidence interval) is plotted in black. In case of spatial autocorrelation between netting walls (i.e., the catches from nearly located netting walls are more similar than expected by chance), correlation values (y-axis) would approaches to 1, thus netting walls seem to be independent each other irrespective of they are closely located or not within the net. This is confirmed by the fact that the actually observed correlation values are within the envelope delineating the 95% envelope when no spatial autocorrelation exists (i.e., when the netting walls are randomly permuted within the net, thus breaking any possible autocorrelation pattern).

**FigureS2. Comparison of PMF versus MMF discards**

Total discards at the net level after pooling the items attributable to a given netting wall (which remain tangled) and the items attributable at the net level only (due to continuously falling on the deck). Net level discards were standardized by the number of netting walls of the net.

**FigureS3. The Kaplan-Meier survival function for undersized spiny lobsters caught and held in captivity for up to 7 days.**


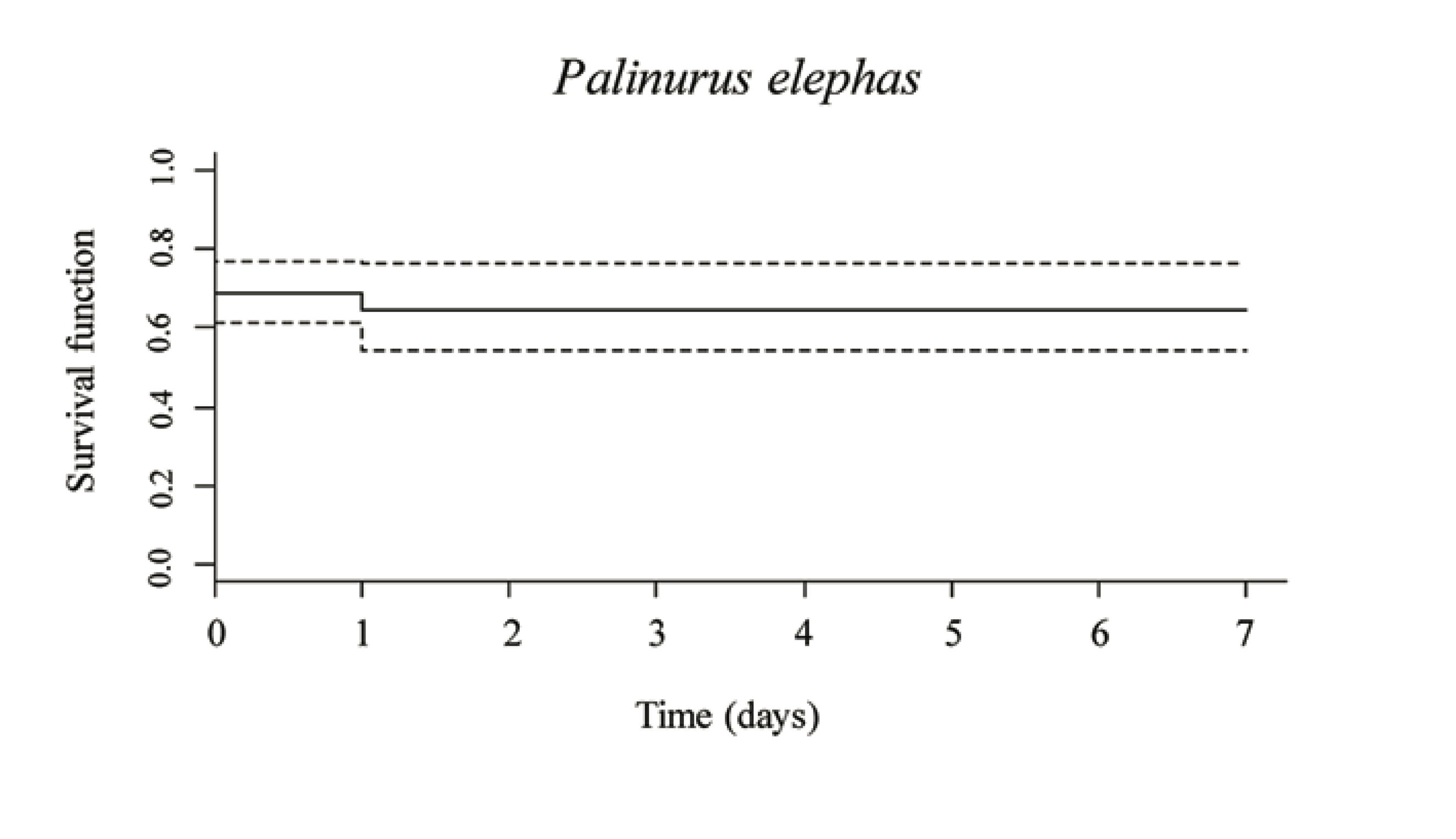


The dashed lines indicate the 95% confidence intervals. This includes observations of immediate mortality, where 30% of animals were dead at arrival on deck.
